# Cancer Stem Cells: Powerful Targets to Improve Current Anticancer Therapeutics

**DOI:** 10.1155/2019/9618065

**Published:** 2019-11-12

**Authors:** Rayana L. Bighetti-Trevisan, Lucas O. Sousa, Rogerio M. Castilho, Luciana O. Almeida

**Affiliations:** ^1^Laboratory of Tissue Culture, Department of Basic and Oral Biology, University of Sao Paulo School of Dentistry, Ribeirao Preto, SP 14040-904, Brazil; ^2^Laboratory of Markers and Cell Signaling of Cancer, Department of Clinical Analyses, Toxicology and Food Sciences, University of Sao Paulo School of Pharmaceutical Sciences, Ribeirao Preto, SP 14040-903, Brazil; ^3^Laboratory of Epithelial Biology, Department of Periodontics and Oral Medicine, University of Michigan School of Dentistry, Ann Arbor, MI 48109-1078, USA

## Abstract

A frequent observation in several malignancies is the development of resistance to therapy that results in frequent tumor relapse and metastasis. Much of the tumor resistance phenotype comes from its heterogeneity that halts the ability of therapeutic agents to eliminate all cancer cells effectively. Tumor heterogeneity is, in part, controlled by cancer stem cells (CSC). CSC may be considered the reservoir of cancer cells as they exhibit properties of self-renewal and plasticity and the capability of reestablishing a heterogeneous tumor cell population. The endowed resistance mechanisms of CSC are mainly attributed to several factors including cellular quiescence, accumulation of ABC transporters, disruption of apoptosis, epigenetic reprogramming, and metabolism. There is a current need to develop new therapeutic drugs capable of targeting CSC to overcome tumor resistance. Emerging in vitro and in vivo studies strongly support the potential benefits of combination therapies capable of targeting cancer stem cell-targeting agents. Clinical trials are still underway to address the pharmacokinetics, safety, and efficacy of combination treatment. This review will address the main characteristics, therapeutic implications, and perspectives of targeting CSC to improve current anticancer therapeutics.

## 1. Introduction

Despite the massive amount of research and rapid development of new therapeutic strategies during the past decade, cancer remains a significant public health problem being the second most common cause of death worldwide. It was estimated a total of 18.1 million new cases of cancer in 2018 and 9.6 million deaths worldwide [1].

The carcinogenesis process is driven by a multistep process initiated by the accumulation of successive mutations in normal cells. Despite the extensive efforts in understanding the signaling pathways that control the process of carcinogenesis, and the therapeutic strategies capable of targeting altered signals, the development of new strategies capable of halting cancer progression remains a challenge. Therapy resistance and tumor relapse are frequently observed for most of the malignancies, and they seem to be driven by the cellular heterogeneity that allows drugs to effectively eliminate some, but not all, malignant cells [2].

Malignant tumors are complex systems composed of tumor cells and normal cells of host tissue with different stromal cells, which help to build the phenotypic heterogeneity and malignancy of solid tumors [3]. Intertumor heterogeneity is responsible for the tumor individuality and the difficulty to establish a molecular signature for groups of tumors [4, 5]. Besides, intratumor heterogeneity presents a distinct molecular signature in every single patient. The genetic trail of each tumor directly reflects tumor progression, resistance to therapy, and recurrences damping the efficacy of current therapies [6]. Moreover, tumor heterogeneity is, in part, controlled by a small population of tumor cells presenting self-renewal properties known as cancer stem cells (CSC) [7].

CSC display high metastatic potential and contribute to the resistance to conventional anticancer therapy. CSC are relatively rare tumor cells that can self-renew and give rise to the tumor cell heterogeneity that characterizes the complex architecture of tumors. CSC have been identified in various human cancers such as germ cell cancers [8], leukemia [9], breast cancer [10], brain cancer [11], colon cancer [12], pancreatic cancer [13], melanoma [14], head and neck [15, 16], and several other tumors [17, 18]. The presence of CSC in different tumors suggests a common trend in cancers and thereby a potential target to therapy [19].

The concept of CSC was first introduced in 1928, in which studies recognized similarities among cancer progression and the development of an embryo, originating the embryonic model of tumor origin [20]. However, only in 1991, the CSC model was demonstrated in leukemia, showing the existence of a small population of cells capable of initiating leukemia [9]. Subsequent investigations on different tumors have shown that not all cells in a tumor were endowed with the capacity to propagate efficiently. In fact, it was shown that only CSC have tumorigenic activity that enables them to form tumors when transplanted into animals and can be the source of all tumor cells present in a malignant tumor [21, 22]. It was only later in 2005 that the existence of CSC population was demonstrated *in vivo* for the first time. Using fully penetrant transgenic mouse models in melanoma [14] and breast [23], intestine [24], and brain cancers [25], researchers identified a group of stem/progenitor cells as cancer-initiating cells and obtained insight into the behavior of these tumors. CSC display resistance to apoptosis, and they are capable of evading the immune system. CSC have similar physiological properties as normal stem cells, like self-renewal, differentiation, and indefinite proliferation ability which might be the main cause of tumor progression [26]. They also can assume a quiescent state, which contributes to the resistance to therapy, and later, they can proliferate and differentiate through asymmetric divisions, promoting recurrence and distant metastases [18].

Current therapies fail to cure metastatic solid tumors; even though they have cytotoxic and/or cytostatic effects over cancer cells, their ability to eliminate cancer stem cells remains poorly understood. The knowledge acquired on CSC biology in recent years supports better detection and isolation and improved therapeutic target of these cells [27]. As a result, the development of new combined therapies, including the use of epigenetic modifiers, stemness inhibitors, and CSC surface markers and immunotherapy are currently in clinical trial [18].

**Figure 1:**
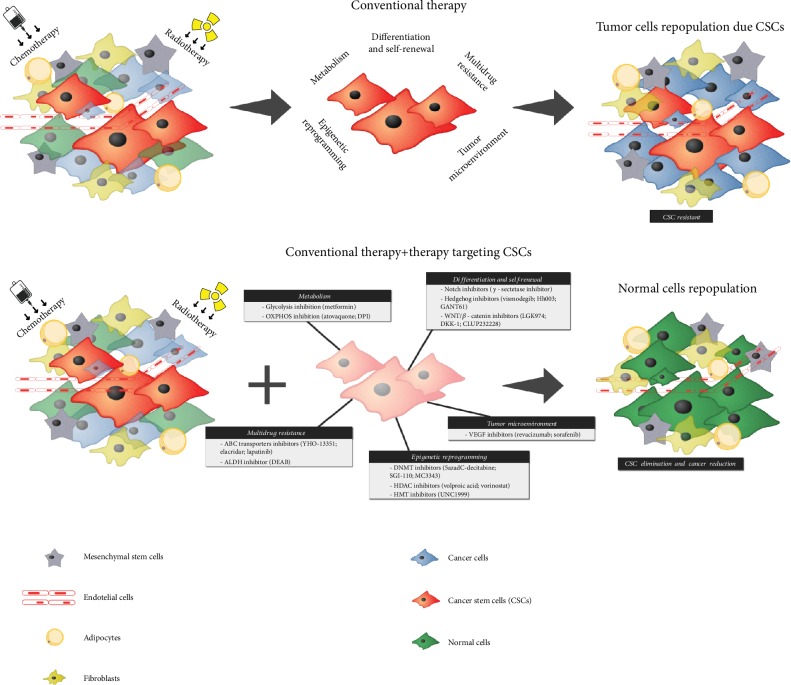
Targeting cancer stem cells signaling to overcome resistance of current anticancer therapy. While the conventional chemotherapy and radiotherapy eliminate more differentiated cancer cells, specific phenotypes of CSC (i.e., multidrug resistance, epigenetic reprogramming, and tumor microenvironment protection) allow them to evade the conventional treatments and avoid the cell death. Once CSC population accumulates, after conventional therapy, they start to regrowth the tumor promoting cancer recurrence. To avoid recurrence, a more efficient therapeutic regimen purposes the administration of new drugs that directly target CSC metabolism, self-renewal, differentiation or other stem cell particularities to disrupt CSC, concomitant with conventional therapies to eliminate differentiated cells.

In this review, we will focus on the most recent therapeutic strategies in development targeting CSC and its mechanisms associated with chemo- and radioresistance (Figure 1).

## 2. Cancer Stem Cells and Resistance to Therapy

Probably, one of the challenges of modern medicine is efficiently managing and treating solid tumors. Surgery was the first tool available since 1809 when Ephraim McDowell removed an ovarian tumor providing evidence that some tumors could be cured by surgery. Radiation was developed to fight cancer in 1950. Radiotherapy uses high-energy ionizing radiation to inhibit tumor growth, leading to cell death in some cases [28, 29]. Advances in radiation therapy lead to marginal success in cancer management with the cure of one-third of all patients receiving combined radiation and surgery [30]. In the early 1900s, Paul Ehrlich started to develop drugs to treat infectious diseases, introducing the term chemotherapy. In cancer, chemotherapy works by killing or slowing down tumors by targeting dividing cells, yet nonproliferating cells are often left behind [31]. The most common drugs, nitrogen mustards, and platinum agents represent more than half of the approximately 4000 open clinical trials using DNA crosslinking agents worldwide. However, only 12% of these represent late-phase trials focused on relapsed or recurrent cancers [32]. Currently, chemotherapy is the therapy of choice for inoperative tumors and preoperative pharmacotherapy [33]. Some examples of these drugs include temozolomide used as a standard treatment for glioblastoma [34]; docetaxel used to treat metastatic prostate cancer [35]; and platinum derivatives employed in the treatment of several nonoperative cancers such as lung [36], head and neck [37], and colorectal cancers [38]. Combined chemotherapy and radiotherapy have been the therapy of choice for many solid tumors capable of improving overall survival [39].

The population of CSC might be responsible for the lack of success of therapeutic strategies currently available [40, 41]. Either single or combined treatments target only the bulk of the tumor, and the elevated rates of tumor recurrences are attributed to the accumulation of CSC [42, 43]. The resistance mechanisms endowed by CSC are attributed to several factors including a transient cellular quiescence, the accumulation of ABC transporters, and disruption of cellular apoptosis [18]. Chemoresistance associated with CSC is observed in colorectal and ovarian cancer, where activation of the serine-threonine kinase named Aurora-A is involved in resistance to apoptosis and tumorigenicity maintenance [44]. In salivary gland tumors, administration of cisplatin is associated with accumulation of CSC and the chemoresistance is controlled by epigenetic modifications [45, 46]. In glioblastoma, CSC contributes to the resistance to temozolomide through increased expression of the repair protein MGMT, ABC transporter BCRP1, and several antiapoptotic proteins [47].

There is growing evidence that CSC are also innately resistant to radiation, by stimulating the repair of DNA damaged, redistributing the cells in the cell cycle, increasing activation of the DNA damage checkpoint, and repopulating and reoxygenating areas of hypoxia in the tumor [48]. CSC contribute to glioma radioresistance through promptly activating DNA damage response, which increases DNA repair, thus promoting cell survival [49]. In breast cancer, radioresistance of CSC is associated with lack of oxidative stress due to their increased ability to eliminate free radicals and the activation of DNA repair [50]. Mammary CSC can also be enriched after radiotherapy through the activation of WNT/*β*-catenin signaling that promotes self-renewal [51]. CSC from mucoepidermoid carcinoma contributes to radioresistance due to the activation of NF*κ*B signaling [52]. Overall, the understanding of the role of CSC in tumor formation and maintenance is the key to improve the new therapeutic technologies currently developed to improve short- and long-term survival.

## 3. Strategies to Target Cancer Stem Cells

### 3.1. Differentiation and Self-Renewal

CSC share many characteristics with normal stem cells. Several studies correlate the origin of CSC with normal stem cells that underwent oncogenic transformation due to mutation-induced genome reprogramming and epigenetic deregulations [53]. The molecular signaling that governs normal stem cell homeostasis is highly regulated. Many of these controlling pathways are abnormally activated leading to the loss of self-renewal and proliferation control, along with increased survival, and differentiation of CSC [54, 55]. Three main signaling pathways including Notch, Hedgehog, and Wnt sustain survival, proliferation, and the balance between differentiation and CSC self-renewal [56].

In normal stem cells, Notch receptor is involved in cell proliferation, differentiation, and apoptosis. Once activated, Notch is translocated to the nucleus, initiating cell transduction and increased transcription. Deregulated Notch signaling leads to abnormal cell proliferation and decreases cellular differentiation and apoptosis and has been implying in the maintenance of CSC in cancer [57, 58]. The use of Notch inhibitors as a single agent or in combination with chemotherapeutic agents has been applied in the treatment of cancer. Capodanno et al. [59] interfered with the Notch pathway by using gamma-secretase inhibitor (GSI) in combination with 5-fluorouracil (5-FU) and observed a decrease in clonogenicity and tumorigenicity of CSC. In glioblastoma, activation of Notch signaling promotes resistance to temozolomide and radiotherapy, accumulating CSC and activating angiogenesis [60]. The administration of Notch-target gamma-secretase inhibitors in combination with farnesyltransferase inhibitors was efficient in sensitized CSC to radiotherapy [61]. Nanoparticles carrying gamma-secretase inhibitors efficiently reduced self-renewing of CSC and suppressed tumor growth in breast cancer [62].

The Hedgehog (Hh) signaling plays an important role in tissue homeostasis, control of cell polarity, and regulation of embryonic development [63]. Uncontrolled activation of the Hedgehog pathway is associated with many cancers [64–66], and it was recently involved in the chemoresistance phenotype due to the accumulation of CSC [67, 68]. Small Hh inhibitors, such as vismodegib (antagonist of smoothened receptor), can suppress cell proliferation and tumor growth [69]. Chen et al. [70] analyzed the effect of Hh003, a new inhibitor of the smoothened receptor (SMO), demonstrating that Hh003 activates caspase-8, inducing apoptosis in colorectal cancer and promoting the inhibition of tumor growth *in vivo*. The Hh signaling is also related to the maintenance and accumulation of glioma CSC, and the use of the inhibitor diminished proliferation, survival, and self-renewal of CSC, reducing the expression of SOX2, required for stem-cell maintenance [71]. Using a low-throughput drug-screening platform, Balic et al. [72] found that chloroquine efficiently eliminated pancreatic CSC inducing antiproliferative effects via reduction of SMO, improving standard chemotherapeutic regimens. GANT61, a potent inhibitor of the noncanonical Hh pathway reduced CSC in breast cancer cell lines, inhibiting tumor growth through G1 cell cycle arrest and induction of apoptosis [73]. GANT61 also suppressed CSC from pancreatic cancer showing enhanced activity in inhibiting tumor progression when in association with rapamycin [74]. PF-04449913 (PF-913) is a selective, small-molecule inhibitor of SMO. In acute myeloid leukemia (AML), PF-913 decreased CSC population, modulating self-renewing properties and cell cycle progression, and it also sensitizes AML to chemotherapy [75].

The Wnt/*β*-catenin pathway is fundamental in the regulation of tissue and stem cell self-renewal [76], but several studies demonstrated the association of Wnt/*β*-catenin and cancer. In the canonical pathway, *β*-catenin is translocated to the nucleus, leading to activation of transcription factors that control the expression of target genes. In the noncanonical Wnt pathway, signals are transmitted through GTPase proteins promoting changes in cellular polarity [77]. Inhibition of Wnt signaling using either the porcupine inhibitor LGK974, short hairpin RNA (shRNA) targeting *Porcn* or recombinant DKK-1 (a Wnt antagonist), reduced CSC, tumor growth, and the proliferative potential of the lung cancer cells, leading to improved survival of patients [78]. Patients with metastatic colorectal cancer have an increased level of blood progastrin, a tumor-promoting peptide essential for self-renewal of colon CSC, which is also a direct target of *β*-catenin/TCF4. Antibodies against progastrin efficiently decrease self-renewal of CSC sensitizing colorectal patients to chemotherapy [79]. CWP232228 is a small molecule that potently inhibits Wnt signaling by antagonizing the binding of *β*-catenin to T-cell factor (TCF) in the nucleus, which downregulates a subset of Wnt/*β*-catenin-responsive genes. Administration of CWP232228 reduces the accumulation of CSC and reversed radioresistance phenotype in breast cancer [80]. In epithelial ovarian cancer, resistance to platinum-based therapy is associated with the maintenance of CSC via upregulation of the Wnt/*β*-catenin pathway, and the treatment using niclosamide, a salicylamide derivative, promoted significant inhibition of proliferation and eliminated CSC by significantly decreasing the expression of proteins from the Wnt/*β*-catenin pathway [81].

Another pathway involved in the self-renewal and proliferation of normal stem cells and cancer stem cells is the JAK-STAT pathway. Usually, this pathway is activated by several ligands, which phosphorylate and activate JAK following by the recruitment of the transcriptional factor STAT [82]. Nevertheless, in oncogenic situations, the JAK-STAT pathway is unusually activated and contributes to tumor development through the accumulation of CSC. Leng et al. [83] found that the JAK/STAT pathway plays a vital role in the accumulation of glioblastoma stem cells, mediating the resistance to temozolomide and administration of JAK inhibitor AG490, improves the current chemotherapy, inhibiting tumor growth and proliferation of CSC. JAK-STAT signaling is constitutively activated in prostate CSC and blocking STAT3 activation using LLL12, a molecule that inhibits the phosphorylation of STAT3 monomer which suppresses CSC and diminishes tumorigenicity [84]. Celecoxib, a selective COX-2 inhibitor, has been shown to potentially reduce STAT3 phosphorylation. In medulloblastoma, the administration of celecoxib suppressed the CSC-like properties and enhanced the radiotherapy efficiency [85].

### 3.2. Multidrug Resistance

Among several drug resistance mechanisms used by cancer cells to evade chemotherapy, the ability of tumor cells to increase cellular efflux of administered drugs retaining low intracellular levels is unique. The ATP-binding cassette transporter (ABC transporter) is a multidrug efflux pump that can transport substrates and drugs across cellular membranes using ATP hydrolysis. This mechanism is strongly present in CSC which have enhanced anticancer drug efflux, by presenting overactivation of several ABC transport genes, including ABCB1, ABCG2, and ABCC1 [86]. The accumulation of ABCG2 in glioma stem cells (GSC) resistant to demethoxycurcumin (DMC) was evaluated by Chen et al. [87]. It was observed that the suppression of ABCG2 induced apoptosis and increased the levels of reactive oxygen species (ROS) in GSC treated with DMC, overcoming chemoresistance. YHO-13351 is a potent inhibitor of ABCG2, and its combination with irinotecan effectively targets CSC from cervical carcinoma preventing resistance and tumor relapse [88]. Elacridar is a third-generation competitive inhibitor of ABCB1 that reverses multidrug resistance of lung cancer, sensitizing cancer stem cells to docetaxel [89]. In breast cancer, ABCC1 and ABCC3 transporters are implicated in multidrug resistance and are increased after chemotherapy. Diminished expression of ABCC1 and ABCC3 transporters leads to the reduction of CSC population promoting the retention of therapeutic drugs inside cancer cells [90]. Lapatinib is a small molecule that acts at the ATP-binding site of tyrosine kinase domains, and its administration increases the accumulation of chemotherapeutic agents in tumors with multidrug resistance through the inhibition of ABCB1 and ABCG2 in CSC from metastatic breast cancer [91].

ALDH is a major marker of CSC in different cancers. This enzyme is involved in cellular detoxification of normal stem cells through the catalysis of aldophosphamide oxidation and used by CSC to neutralize chemotherapeutic drugs as part of a multidrug resistance process [92]. DEAB (N,N-diethylaminobenzaldehyde) is a specific ALDH inhibitor that increases the sensitivity of breast cancer CSC presenting high enzymatic levels of ALDH enzymes to paclitaxel and epirubicin [93]. ALDH1A3 is highly expressed in CSC from malignant mesothelioma, and the repression of STAT3-NF*κ*B signaling diminishes ALDH1A3 accumulation sensitizing cancer cells to pemetrexed and cisplatin treatments [94]. ALDH1 activity is also increased in colon cancer stem cells with multidrug resistance phenotype, and the pharmacological inhibition of ALDH1 using DEAB and the molecular inhibition using interference RNA sensitized the cells to capecitabine and 5-fluorouracil [95].

### 3.3. Epigenetic Reprogramming

Recent advances in gene reprogramming using the Yamanaka factors [96] to induce pluripotency of adult cells suggest that cellular dedifferentiation may play a role in CSC formation development. Moreover, it became clear that cellular reprogramming involves dramatic methyl modifications in CpG islands [97] supporting the concept that epigenetic modifications may also be involved in dedifferentiation of transformed tumor cells [98]. Indeed, epigenetic reprogramming of primary human colon cancer cells using the expression of OCT4, SOX2, Klf4, and c-MYC generated clonogenic CSC with enhanced metastatic potential. The reprogramming of colon tumor cells reduced the methylation of the promoter region of NANOG leading to protein overexpression. Genetic knockdown of NANOG in the reprogrammed tumor cells eradicated their clonogenic potential. With that, it became evident that epigenetic modifications occurring during cellular reprogramming are critical to the generation of CSC [99].

Since the epigenetic modifications are major events capable of inducing cellular reprogramming and modulating stem cell properties in tumors, the elimination of the CSC can be achieved by targeting epigenetic regulators [100]. JMJD3 is one of two histone H3K27me3 demethylases, and it has been reported to participate in the regulation of tumorigenesis. Upregulation of JMJD3 inhibits the expression of the transcription factor Oct4 promoting the suppression of tumor growth and diminishing CSC from breast cancer, suggesting JMJD3 as a potential target to overcome resistance mediated by CSC [101]. Recent observations on the reduced levels of the Androgen Receptor (AR) in prostate CSC due to the hypermethylation of the promoter region of AR provided the mechanistic basis to Tian and collaborators [102] to use decitabine (5-aza-2'-deoxycytidine-5azadC) to inhibit DNA methyltransferases (DNMTs) in prostate tumors. This strategy resulted in the elevation of AR levels and the differentiation of the prostate CSC. In breast cancer, low dose of decitabine encapsulated in nanoparticles in combination with doxorubicin efficiently reduced the population of CSC while improving patient response to therapy and accumulation of apoptotic tumor cells [103]. SGI-110 is a second-generation DNA methyltransferase inhibitor that reduces the stem-like properties of ovarian cancer cells, such as their tumor-initiating capacity, through the global tumor hypomethylation. SGI-110 was also found to sensitize tumor cells to carboplatin and to reexpress genes associated with differentiation [104]. A recent study [105] described how a new DNMT inhibitor (DNMTi) MC3343 was able to block osteosarcoma cell proliferation. MC3343 presents an antiproliferative effect similar to 5azadC, inducing CSC differentiation and osteoblastic maturation. This drug presents synergistic effects with cisplatin and doxorubicin, and the combination results in DNA damage and cell death.

Another class of epigenetic modifiers used to target CSC is the Histone deacetylases (HDACs) inhibitors. The valproic acid was tested in breast cancer cell line resulting in enhanced acetylation of the p21 promoter region and increased protein levels of p21. Also, the valproic acid decreased the level of CD44 antigen originating more differentiated cells. The inhibition of HDACs reprogrammed the CSC to a more differentiated phenotype, which is more responsive to the current chemotherapies [106]. Vorinostat (suberoylanilide hydroxamic acid, SAHA) is an efficient class I and II HDAC inhibitor approved in 2006 for the treatment of lymphoma [107]. Vorinostat specifically triggers autophagy and reduces cell viability via differentiation of glioblastoma stem cells. *In vivo* models revealed that vorinostat effectively reduces tumor growth and induces autophagy through the downregulation of AKT-mTOR signaling [108]. Vorinostat is also effective in reducing CSC from the salivary glands. Administration of vorinostat resulted in the sensitization of adenoid cystic carcinomas and mucoepidermoid carcinomas to cisplatin through the reduction of CSC population [45, 46]. EZH2 is a histone methyltransferase that targets histone H3 at lysine 9 and 27, leading to transcriptional repression. EZH2 inhibitor UNC1999 efficiently eradicates self-renewal of glioblastoma CSC and reduces tumor growth in combination with temozolomide [109].

### 3.4. CSC Metabolism

Normal proliferating cells under regular oxygen conditions (normoxic) obtain energy to maintain homeostasis converting sequentially glucose to glucose-6-phosphate, fructose-6-phosphate, fructose-bisphosphate, and acetyl coenzyme A. In mitochondria, Acetyl CoA enters in the Krebs cycle (or tricarboxylic acid cycle, TCA) to produce nicotinamide adenine dinucleotide (NADH) and flavin adenine dinucleotide (FADH2) molecules. The final balance of OXPHOS generates up to 38 molecules of ATP per glucose molecule. Alternatively, in hypoxic situations, the cytoplasmic glycolytic metabolism is triggered to convert glucose in lactate, generating NADH and two molecules of ATP per glucose [110]. It is well known that normal stem cells rely their metabolism on glycolysis [111–113] due to their reduced number and immature mitochondria, contributing to an environment that generates less reactive oxygen species (ROS). Interestingly, lower levels of ROS are essential for the undifferentiated stem cell phenotype [114].

#### 3.4.1. CSC Glycolytic Metabolism

In nonsmall lung cancer cell lines, Liu et al. [115] observed that CSC presented higher glycolysis rates than differentiated cells. The level of lactate and the level of genes involved in glycolytic metabolism (such as HK-1, HK-2, Glut-1, and PDK1) were higher in the CSC after glucose administration. CSC from breast tumors also showed a higher glycolytic metabolism when compared to non-CSCs [116]. Undifferentiated breast cancer cells (CD49^high^EPCAM^low^) presented lower levels of key enzymes involved in the TCA (ldh1, Aco1, Sdha, and idh3g) when compared to more differentiated cells (CD49^low^EPCAM^High^) [116]. Chen et al. [117] overexpressed NANOG in a mouse model of hepatocellular carcinoma, which led to a decrease in OXPHOS, whereas NANOG knockdown upregulated the OXPHOS genes, reducing glycolytic activity. In oral cancer cells, EGF drives a glucose metabolic reprogramming, and the accumulation of CSC increases the levels of L-lactate through the activation of EGFR/PI3K signaling. The administration of 2-deoxy-D-glucose, a competitive inhibitor of glucose-6-phosphate, efficiently reversed the process diminishing glycolysis and the accumulation of CSC [118]. Metformin, a first-line drug used in the diabetes treatment, was linked to cancer prevention inhibiting cellular transformation and selectively killing breast CSC, which reduces glycolytic and TCA cycle metabolites [119]. In colorectal cancer, metabolic reprogramming of CSC is modulated by the adenylate kinase hCINAP, and the depletion of this enzyme using molecular strategies reverses several CSC phenotypes including self-renewal, EMT, and chemoresistance [120].

#### 3.4.2. CSC OXPHOS Metabolism

OXPHOS metabolism also contributes to the phenotype of CSC. CD34^+^ cells isolated from the bone marrow of patients with Acute Myeloid Leukemia (AML) present low levels of ROS associated with quiescence, self-renewal, and chemotherapy resistance. This CSC (ROS-low) population also presented a lower glycolytic rate when compared to ROS-high population. Still, ROS-low cells were unable to use glycolysis when oligomycin and FCCP inhibited mitochondrial function, indicating that mitochondrial respiration and function (OXPHOS) is crucial for CSC in AML [121]. In epithelial ovarian cancer (EOC), CSC population (CD44^+^CD117^+^) presented low levels of Phospho-Pyruvate Dehydrogenase (pPDH), Pyruvate Dehydrogenase Kinase (PDHK1), and MCT4 (lactate transporter) when compared to non-CSC. The reduced amount of these enzymes that drive the glycolytic metabolism strongly indicates a commitment to the tricarboxylic acid cycle and OXPHOS metabolism for EOC [122].

Metformin decreases the blood glucose levels through the suppression of hepatic gluconeogenesis and increases glucose uptake by skeletal muscle. Metformin interferes in the mitochondrial respiratory chain, activating 5′AMP-activated protein kinase (AMPK), suppressing the activity of mTOR, and reducing the levels p-S6K1. This alteration has been shown to sensitize breast CSC (CD44^High^/CD24^Low^) for radiotherapy [123]. Atovaquone is an FDA-approved antimalarial drug and analog of Co-enzyme Q10 (CoQ10) acting as a potent and selective OXPHOS inhibitor, by targeting the CoQ10-dependence of mitochondrial complex III. In breast cancer, atovaquone has anticancer activity against CSC, inhibiting oxygen consumption and accumulating oxidative stress, which induced apoptosis of CSC population [124]. In a high-throughput drug screening, Ozsvari et al. [125] identified DPI (Diphenyleneiodonium chloride), a compound that potently blocks mitochondrial respiration by inhibiting flavin-containing enzymes (FMN and FAD-dependent), as a new potential therapeutic agent against breast CSC. DPI induced a chemoquiescence phenotype that effectively inhibited the propagation of CSC.

### 3.5. Tumor Microenvironment and CSC

Shelter, perhaps, is the most important function of the tumor microenvironment (TME) for CSC. The TME is composed basically of extracellular matrix, stromal cells (including fibroblasts, mesenchymal, endothelial, and immune cells), and an intricate network of signaling molecules. The whole structure and components of the TME provide ideal conditions for the maintenance of the CSC capacities to unlimited self-renewal, proliferation, differentiation, and generation of the heterogeneous population that characterizes the cancers. Besides, over the past years, this harborage has been implicated in the cancer therapy resistance [126].

#### 3.5.1. Extracellular Matrix

The TME three-dimensional component, noncellular and composed of glycosaminoglycans, collagens, metalloproteases, hyaluronic acid, polysaccharides, glycoproteins, proteoglycans, and a diversity of other proteins, is called extracellular matrix (ECM). Virtually, this well-organized structure is present in all tissues and provides physical stability and an infinitude of signaling that controls cellular survival, proliferation/growth, differentiation, and migration [127].

The balance of all ECM components is closely involved in CSC survival and therapy resistance. One remarkable example of the crosstalk among ECM components and the cancer biology/resistance was described in colorectal liver metastasis. It was observed that the benefits of pretreatment of patients with anti-VEGF (bevacizumab) are limited to a few months because of acquired resistance. Metastatic colorectal liver patients and mouse models were treated with anti-VEGF therapy, and the deposition of hyaluronic acid and glycosaminoglycan within the tumors was increased. This situation enhanced the stiffness of the liver tumors, compromising intratumor perfusion and leading to a reduction in intratumor delivery of anticancer drugs [128]. If the tumor mass is not satisfactorily reached by the antitumor drug, the ECM acts as a shield for the CSC. In a hepatocellular carcinoma model, the pharmacological inhibition of hyaluronic acid using 4-methylumbelliferone promoted a decrease in the levels of CSC markers and stimulated apoptosis while decreased tumor growth and metastasis [129].

#### 3.5.2. Stromal Cells

*(1) Fibroblasts*. In a tumor context, the cancer-associated fibroblasts (CAFs) give support to the initiation, development, and maintenance of transformed cells [130]. Nair et al. [131] cultivated mouse-induced stem cells (miPS cells) using conditioned culture medium from two different breast cancer cell lines. The miPS cells differentiated into CSC, presenting high levels of CD133 and epithelial carcinoma marker (EPCAM). These CSC were induced to differentiate and originated myofibroblast-like cells. Myofibroblast-like cells showed a high level of CAF markers (fibroblast-specific protein (FSP1), *α*-smooth muscle actin (*α*-SMA), stromal-derived factor-1 (CXCL12), and transforming growth factor (TGF*β*1)) and were observed that CAFs give support to the CSC self-renewal. In breast, CSC and CAFs interact through Hedgehog signaling, in which CSC secrete SHH ligand regulating CAFs via paracrine activation of Hedgehog. In turn, CAFs secrete a factor that stimulates CSC self-renewal, and the Hedgehog inhibitor vismodegib reduces CAFs and CSC accumulation by slowing tumor formation [132].

*(2) Adipocytes*. The adipose stem cells (ASCs) are one of the most abundant cells present in the tumor microenvironment. They secrete several molecules with crucial impact in inflammation and angiogenesis, such as leptin, Interleukin-6 (IL-6), and Platelet-derived growth factor (PDGF) [133, 134]. ASC is derived of stem cells from mesenchymal lineage, and they are capable of differentiating into adipocytes, myocytes, osteocytes, or chondrocytes, which draw attention to their therapeutic potential for treating disorders or even their involvement in the development of diseases such as cancer [135]. Conditioned culture media obtained from obese-altered ASC were capable of generating MCF7 breast cancer cell metastasis through the upregulation of ABCB1 and SERPINE1 genes [136]. In prostate cancer, ASC stimulates adipocyte-induced prostate tumor growth through the activation of STAMP2 expression [137]. A recent study demonstrated that coculturing ASC pretreated with paclitaxel inhibited breast cancer proliferation and survival *in vitro* and *in vivo*, indicating the potential of this strategy to avoid cancer relapse [138]. Regarding CSC, adipocytes from prostate cancer niche induce CSC accumulation through the stimulation of paracrine factors such as cathepsin B [139].

*(3) Mesenchymal Cells*. Mesenchymal stem cells (MSCs), first isolated from the bone marrow and found in several other tissues, present the plasticity in differentiating into the cartilage, bone, and fat cells, among others [140]. It has been described in TME and in the neoplastic niches that MSC support cancer cell proliferation, protect them against ROS, and stimulate the epithelial-mesenchymal transition (EMT), inducing transformed cells to get more metastatic phenotype [141]. MSC in TME is also associated with resistance to chemotherapy. Using two different mouse tumor models (colon carcinoma and Lewis lung carcinoma), Roodhart et al. [142] observed that the intravenous injection of MSC induced resistance to various chemotherapeutics and this MSC-induced resistance was dependent on the activation of the mesenchymal cells with platinum-based drug.

*(4) Endothelial Cells*. As basic vasculature units, endothelial cells (EC) have the function not only as structural entities, giving rise to the system that provides oxygen and nutrients to the tumors. EC also work as mediators of signaling in the TME, contributing to survival, self-renewal, EMT, and metastatic phenotype of the cancer cells [143]. It has been observed that the endothelial nitric oxide synthase (eNOS) expression and activity are higher in glioma cells neighboring the vascular endothelium. In this sense, Charles et al. [144] described that Nitric Oxide (NO) activates the Notch pathway in glioma stem cells located close to the endothelium and this activation of Notch signaling accelerates the onset of glioma and tumor formation in murine model increasing CSC.

#### 3.5.3. Immune Cells

Failures of the immune system to identify and eliminate transformed cells have been described as a major cause for cancer development [145]. CSC can modify the immune cells located in the TME to maintain an enabling environment. This situation was observed by studying CSC in a rat glioma model. The authors observed that CSC induced bone marrow-derived monocytes to differentiate into tumor-associated macrophages (TAM). The TAM CD11c^high^ showed protumor activity, and its generation was attributed to the secretion of GM-CSF by the CSC, putting TMA CD11c^high^ as a candidate for therapy to destabilize CSC niches [146]. Immunologic malfunction also occurs because CSC acquires abilities to escape from the immune system. After CD8^+^ T cells were incubated with CSC (CD44^+^) or more differentiated cells (CD44^−^) isolated from head and neck squamous cell carcinoma (HNSCC), the authors observed that the CD8^+^ T cells stimulated with CD44^+^ cells produced less IFN*γ* when compared to CD44^−^, showing that CSC present less immunogenicity and this feature favors a scape from the immunologic system [147]. Understanding the molecular mechanisms that lead CSC to escape from the immune system is perhaps one of the most important tasks to be done in the study of cancer.

## 4. Combination Treatment as a Strategy to Eliminate CSC and Reverse Chemo- and Radioresistance

After diagnosis, a tumor harbors at least ten or more subclones that are resistant to monotherapy. Consequently, single-targeted agents are unlikely to effectively kill all types of tumor cells due to tumor heterogeneity [148]. Besides, many tumors develop resistance post treatment, urging the discovery of novel therapeutics. Furthermore, identifying agents that can be administrated in combination is a strategy to avoid or overcome treatment failure by minimizing resistance and preventing further progression to metastatic disease [32].

The accumulation of data from cancer cell lines and animal models strongly supports the potential benefits of combination treatment. Several clinical trials are currently under investigation to explore efficacy, safety, and pharmacokinetics of combining chemotherapeutic drugs with anti-CSC agents. A summary of clinical trials using combination treatment is displayed in Table 1 (according to the United States National Cancer Institute ClinicalTrials.gov). NCT01876251 is a phase I clinical trial that evaluates maximum tolerated dose, safety, pharmacokinetics, and antitumor activity of the combination between PF-03084014 and docetaxel in patients with breast cancer. PF-03084014 is a reversible, non-competitive, selective gamma-secretase inhibitor that blocks NOTCH signaling, interfering with the survival of CSC. Sixteen percent of the patients present a partial response to the treatment [149]. A successful trial evaluating the inhibition of NOTCH signaling to target CSC is in phase IB (NCT01189968) and associates demcizumab combined with carboplatin and pemetrexed as first-line treatment for patients with previously untreated advanced-stage nonsquamous non-small-cell lung cancer (NSCLC). Demcizumab is an IgG2 humanized monoclonal antibody directed against delta-like ligand 4-Notch (DLL4-Notch) signaling, which contributes to chemoresistant CSC and tumor vasculature. The primary objective of this trial was to determine the maximum tolerated dose of the combination and define safety, rates of immunogenicity, preliminary efficacy, pharmacokinetics, and pharmacodynamics. Fifty percent of the patients evaluated had objective tumor responses, which was numerically higher than the expected with the standard pemetrexed-platinum chemotherapy, and the combination was recommended to phase II study [150]. Another successful clinical trial targets CXCR1, which is an actionable receptor selectively expressed by breast cancer stem cells (BCSC). CXCR1 is a receptor for CXC ligand 8 (CXCL8; formerly interleukin 8), a proinflammatory chemokine implicated in metastasis and progression of multiple malignancies. Reparixin is an allosteric inhibitor of CXCR1/2 that demonstrates activity against BCSC in xenograft models. A phase IB clinical trial examined dose, safety, and pharmacokinetics of paclitaxel plus reparixin therapy, and explored effects of reparixin on BCSCs in metastatic breast cancer patients (NCT02001974). The combination seemed to be safe and tolerable, and a 30% response rate was recorded with a reduction of paclitaxel doses, suggesting further studies in a randomized phase II trial [151].

Despite the fact that CSC are resistant to radiotherapy [39, 152] and are often found accumulated after radiotherapy [153, 154], few clinical trials focus on targeting CSC to overcome radioresistance. NCT02039778 was a phase II study focusing on targeting the CSC niche that resides in the lateral periventricular regions of the brain, using irradiation combined to temozolomide in patients with high-grade glioma. Another phase II study aimed to combine fursultiamine plus irradiation and chemotherapy (NCT02423811). Fursultiamine (also known as thiamine tetrahydrofurfuryl disulfide, TTFD) is a derivative of vitamin B used as a nutrition supplement that efficiently suppresses CSC markers OCT-4, SOX-2, and NANOG expression and decreased ABCB1 and ABCG2 in tumor spheres of esophageal carcinoma. NCT01119599 is a phase I trial of RO4929097 (gamma-secretase/Notch signaling pathway inhibitor) in combination with standard radiotherapy and temozolomide for newly diagnosed malignant glioma. Unfortunately, none of these studies have provided significant results yet (source: United States National Cancer Institute ClinicalTrials.gov).

Besides the growing amount of clinical trials ongoing, several preclinical studies are currently investigating new approaches to target CSC and reverse resistance. CBL0137 is an inhibitor of FACT (histone chaperone facilitates chromatin transcription) highly expressed in CSC. De et al. [155] explored the ability to combine cisplatin and CBL0137 to target small cell lung CSC and to reduce tumor size. CBL0137 alone efficiently diminished CSC, and the combination of cisplatin and CBL0137 significantly reduced the growth of PDX tumor models and also the growth of a syngeneic mouse SCLC tumor. BBI608 is a small molecule that inhibits STAT3, a critical mediator for the maintenance of cancer stemness. BBI608 suppresses cancer relapse, progression, and metastasis. BBI608 inhibited stemness gene expression, depleted CSC, and overcame cisplatin resistance in NSCLC [156]. Cell surface vimentin (CSV) is an effective target to reduce CSC. Unlike intracellular vimentin, which is found in both cancer cells and normal mesenchymal cells, CSV is tumor-specific and is accumulated in CSC from glioblastoma. A novel monoclonal antibody 86C, which recognizes CSV from cancer stem cells, induces apoptosis and overcomes temozolomide resistance in glioblastoma, increasing cell death. Eighty-six C administration reduced the dose of temozolomide required to eliminate tumor cells reducing toxicity [157].

Regarding radioresistance, ongoing preclinical studies have been exploring DNA damage response and apoptosis signaling of CSC to sensitize these cells to radiotherapy. For instance, Timme et al. [158] evaluated the small molecule VX-984 as a radiosensitizer of glioblastoma CSC. VX-984 is an inhibitor of the phosphorylation of DNA-PK, an essential component of the NHEJ repair pathway. VX-984 significantly enhanced the antitumor effect of irradiation in glioblastoma CSC and orthotopic GBM models. PARP protein detects the presence of DNA damage and activates signaling pathways that promote appropriate cellular responses. Thus, PARP inhibitors (PARPi) have emerged as potential radiosensitizing agents. Lesueur et al. [159] investigated the preclinical efficacy of talazoparib, a new PARPi, in association with radiation in reducing CSC population of glioblastomas. The combination drastically reduced accumulation of CSC *in vitro*. Furthermore, talazoparib combined with irradiation induced a prolonged G2/M block decreasing proliferation. Afatinib is a pan-EGFR inhibitor that radiosensitizes head and neck squamous cell carcinoma blocking the accumulation of CSC, inhibiting DNA repair machinery, and inducing apoptosis. Combination of afatinib plus irradiation efficiently decreased the tumor volume in xenograft models [160].

## 5. Conclusions

The journey of cancer therapy is far from the end, and major efforts are necessary to develop effective treatments. CSC population has been presenting resistance to most chemotherapy and radiotherapy regimens currently in use. CSC are continually evolving, constituting functionally heterogeneous cells that are maintained by the plasticity of its niche. Besides chemo- and radioresistance, CSC are also responsible for cancer initiation, tumor relapse, and metastasis, emerging as the major target for the development of successful therapies. Molecular targets that control CSC have the potential to drive the development of new drugs capable of eradicating and preventing the accumulation of new CSC in patients, which could prevent metastasis and tumor relapse, reducing morbidity and toxicity, and ultimately improving the outcomes in cancer patients. Currently, several cell and animal studies strongly support the potential benefits of combining chemo/radiotherapy with CSC-targeting agents; however, the effectiveness of these strategies to improve therapeutic regimens for resistant cancer patients remains largely unknown. Still, a myriad of CSC-targeting agents have been investigated in preclinical and clinical studies, and as more studies are conducted and completed, we expect to achieve a better understanding about the safety and efficacy of the combination strategies.

## Figures and Tables

**Table 1:** Clinical trials involving combination treatment targeting cancer stem cells to overcome chemo- and radioresistance.

Tumor type	Clinical trial ID	Drug targeting CSC (plus chemo/radiotherapy)
Breast	NCT02876302; NCT02776917; NCT02370238; NCT01868503; NCT01876251; NCT02001974; NCT01372579; NCT01281163; NCT01190345; NCT01118975; NCT00949247; NCT00645333; NCT00524303	Ruxolitinib; cirmtuzumab; reparixin; lapatinib; PF-03084014; eribulin mesylate; MK2206; bevacizumab; vorinostat; MK0752; trastuzumab
Pancreatic	NCT022311723; NCT01195415; NCT01192763; NCT01189929; NCT01051284	BBI608; vismodegib; RO4929097; demcizumab; cyberknife radiation
Ovarian	NCT03632798; NCT03030287; NCT02713386; NCT01579812	Avastin; OMP-305B83; ruxolitinib; metformin
Hematologic	NCT03113643; NCT02730195; NCT01861340; NCT01130688	Azacitidine; pioglitazone; MEDI-551; zileuton
Glioma	NCT03632135; NCT02315534; NCT02039778; NCT01119599	Vincristine; irinotecan; etoposide; imatinib; BBI608; stem cell radiotherapy; RO4929097
Colorectal	NCT03035253; NCT02753127; NCT01189942	OMP-305B83; BBI608; OMP-21M18
Non-small-cell lung	NCT01193868; NCT01189968	RO4929097; demcizumab
Hepatocellular	NCT02279719; NCT01442870	BBI503; BBI608; metformin
Esophageal	NCT02423811	Fursultiamine
Gastrointestinal	NCT02024607	BBI608
Head and neck	NCT01255800	IPI-926
Advanced or metastatic	NCT02722954; NCT02483247; NCT02467361; NCT02432326	Demcizumab; BBI503; BBI608
